# Microvascular decompression: Diversified of imaging uses, advantages of treating trigeminal neuralgia and improvement after the application of endoscopic technology

**DOI:** 10.3389/fneur.2022.1018268

**Published:** 2022-11-09

**Authors:** Gui Yu, Jingxing Leng, Yinghua Xia, Feixiang Min, Hui Xiang

**Affiliations:** ^1^The First Clinical Medical College of Gannan Medical University, Ganzhou, China; ^2^Jiangxi Provincial People's Hospital, Nanchang, China; ^3^Medical College of Nanchang University, Nanchang, China

**Keywords:** endoscopic techniques, imaging, microvascular decompression, trigeminal neuralgia, recurrent

## Abstract

Classical trigeminal neuralgia (CTN) is a unilateral and severe facial pain disease, which seriously affects the patient's quality of life. Microvascular decompression (MVD) is currently the most effective surgical method, and it is the only treatment for the etiology of CTN. Imaging for MVD has been increasingly used, and the advantages and disadvantages of endoscopy-assisted vascular decompression surgery have been controversially debated. In this review, we aimed to discuss the advantages of MVD in the treatment of patients with CTN, the importance of using imaging in disease management, and the improvements of vascular decompression surgery through the application and maturity of endoscopic techniques. Compared with other surgical methods, MVD has more prominent short- and long-term treatment effects. Its selection depends on the accurate discovery of neurovascular compression by preoperative imaging. Moreover, magnetic resonance imaging plays a diverse role in MVD, not only in identifying the responsible vessels but also in determining the prognosis and as a tool for scientific research. The use of endoscopic techniques provides improved visualization of the MVD and additional benefits for vascular decompression surgery.

## Introduction

Classical trigeminal neuralgia (CTN) is a functional neurological disease with clear inducing factors. Women are more likely to experience TN than men. The peak age of the patients is 50–60 years. Trigeminal neuralgia (TN) is characterized by transient paroxysmal tingling in the unilateral (mostly on the right) trigeminal nerve branch area with or without changes in facial sensation. The incidence of TN is around 12.6–27/100,000, and the annual incidence is ~4–13/100,000 (1–3); most cases are sporadic. The pain usually occurs suddenly, and a rapid attack reaches its peak, following which it gradually weakens. The duration varies from several seconds to 2 min, and patients usually recall it exactly (4–7). Drinking water, eating, and even talking can trigger pain; therefore, patients will, among others, have anorexia, refuse to drink water, and refuse to socialize, resulting in a serious decline in the quality of life. Therefore, the impact of this disease on patients is not limited to pain itself but also a reduction in their quality of life and impairment of their mental health, and some patients even tend to commit suicide; thus, this intermittent but extremely painful disease is also known as the “suicide disease” (8). Currently, the main management of the disease is medication; however, an increasing number of studies have found that the long-term outcome of drug therapy is not ideal and that surgical intervention is often required in the later stages of the disease. Microvascular decompression (MVD) is currently the preferred surgical method for CTN, and its principle is closely related to the pathophysiological mechanism of CTN. Teflon is used to push the blood vessels away from the nerve to relieve compression, and satisfactory clinical results can be achieved. Although MVD has been widely adopted, a systematic description is lacking, and some deficiencies of conventional MVD may improve with the development of endoscopic techniques. This review article aims to discuss the advances in MVD for CTN in terms of imaging, surgical efficacy, recurrence management, and endoscopic techniques.

## TN classification

Confirmation of TN classification is essential for treatment, as different types of TN require different treatment modalities. According to the latest international classification, TN is divided into three main types (Table 1):

1. CTN: CTN is caused by compression of the nerve by blood vessels in the root entry area. The responsible blood vessel is mainly the superior cerebellar artery, which causes morphological changes in the adjacent nerve roots, leading to their demyelination and increased excitability (8–10). MVD is primarily used for this type of TN.
2. Secondary TN (STN): This type can be attributed to an identifiable neurological disease such as multiple sclerosis or cerebellar horn tumors. Compared to those with CTN, patients with STN are usually younger and more likely to have partial facial sensory loss and bilateral pain (5, 8–10). The therapeutic effects of drugs and MVD are poor, and treatment mainly targets the primary disease.
3. Idiopathic TN (ITN): This is the unexplained type of TN; iCHD-3 defined ITN as “Trigeminal neuralgia with neither electrophysiological tests nor MRI showing significant abnormalities” (2, 5, 8, 9).

**Table 1:** The international classification of headache disorders, 3rd edition.

**13.1.1 Trigeminal neuralgia**
13.1.1.1 Classical trigeminal neuralgia
13.1.1.1.1 Classical trigeminal neuralgia, purely paroxysmal
13.1.1.1.2 Classical trigeminal neuralgia with concomitant continuous pain
13.1.1.2 Secondary trigeminal neuralgia
13.1.1.2.1 Trigeminal neuralgia attributed to multiple sclerosis
13.1.1.2.3 Trigeminal neuralgia attributed to other cause
13.1.1.2.3 Trigeminal neuralgia attributed to other cause
13.1.1.3 Idiopathic trigeminal neuralgia
13.1.1.3.1 Idiopathic trigeminal neuralgia, purely paroxysmal
13.1.1.3.2 Idiopathic trigeminal neuralgia with concomitant continuous pain

In addition, although paroxysmal pain is a characteristic manifestation of TN, about 30% of patients experience persistent pain, which is currently classified as “TN with persistent pain” (2, 5, 8, 10). Patients with persistent pain are unlikely to respond well to drug therapies (5).

## Imaging

No specific clinical symptoms can distinguish the three types of TN, and their discrimination is mainly dependent on whether the imaging examination reveals a potential neurovascular compression (NVC) or other changes associated with demyelination. Therefore, preoperative magnetic resonance imaging (MRI) plays a decisive role in the classification and selection of treatment methods. Related studies have confirmed the reliability of MRI for MVD surgery (11). High-resolution contrast-enhanced MRI can display and evaluate the location, degree, and range of NVC. The combination of three high-resolution sequences: 3DT2-weighted, 3DTOF-MRA, and 3DT1-Gad, facilitates the detection of NVC (12). Once a significantly responsible vessel is identified by imaging, MVD is selected by the neurosurgeon.

Microstructural changes in nerves at vascular compression sites, such as demyelination, can be revealed using diffusion tensor imaging (DTI) and fiber tractography (8, 13–15). Studies have found that the integrity of the white matter connected to the thalamus-somatic sensory cortex on the affected side of patients with TN is significantly decreased, indicating that the cerebral cortex attempts to adapt to potential pain stimuli, which may be a reactive change in the central response to root entry zone damage (13). DTI and voxel-based morphometry were used to study changes in the central nervous system (CNS) structure in patients with CTN. Compared to the brain regions of patients in the control group, those of patients with TN, including the anterior cingulate cortex, insular cortex, somatosensory cortex, hippocampus, premotor area, temporal lobe, and corpus callosum, hadextensive gray matter volume and white matter integrity deterioration. These changes may lead to increased pain intensity, depression, and anxiety in patients with TN, suggesting that the development of TN may cause changes in the brain structures involved in pain and emotional regulation (13, 16–19). Alternatively, these changes may be the result of the CNS gradually adapting to the long-term pain stimuli (13). The abnormal structural and functional patterns observed in the CNS of patients with CTN may provide a better understanding of its pathophysiology.

Furthermore, imaging studies have reported that effective surgical treatment for patients will often reverse the neurological abnormalities, and even some CNS changes will return to healthy levels (14, 20). However, later in the course of the disease, the presence of distal trigeminal atrophy on MRI may suggest that irreversible pathological changes have already occurred which are related to poor prognosis after MVD (21). Some scholars believe that the volumes of the hippocampus and trigeminal nerve can also predict the outcome of surgery (16). In addition, extensive gray matter volume reduction was observed in the CNS of patients with CTN, which may play a role in the long-term changes in CTN, such as aggravated pain, drug resistance, and a prolonged course of the disease (18, 19). Overall, imaging studies support the notion that the CNS is involved in the course of TN. Combining the above results, implementing MVD early in the disease course may be beneficial for patients with CTN.

In conclusion, preoperative MRI can determine whether to select MVD by assessing whether there is NVC and its vascular type and course, and it can help predict the prognosis of MVD. In addition, it can also be differentiated from other diseases, such as STN caused by cerebellopontine angle tumor, multiple sclerosis, or schwannoma. Preoperative differential diagnosis is an important indicator of MVD. Therefore, using MRI as part of the early examination of patients with TN can rule out secondary causes of TN and determine the treatment plan.

## MVD

Currently, medication is the first-line treatment for TN (22, 23). However, long-term pain control by drug therapy is not ideal, and studies have reported a series of side effects of long-term medication, including vertigo, lethargy, nausea, diplopia, and ataxia (24, 25). Therefore, surgical treatment should be considered when drug treatment is ineffective or unacceptable side effects occur. MVD is currently the most effective surgical method (23, 26, 27).

**Figure 1:**
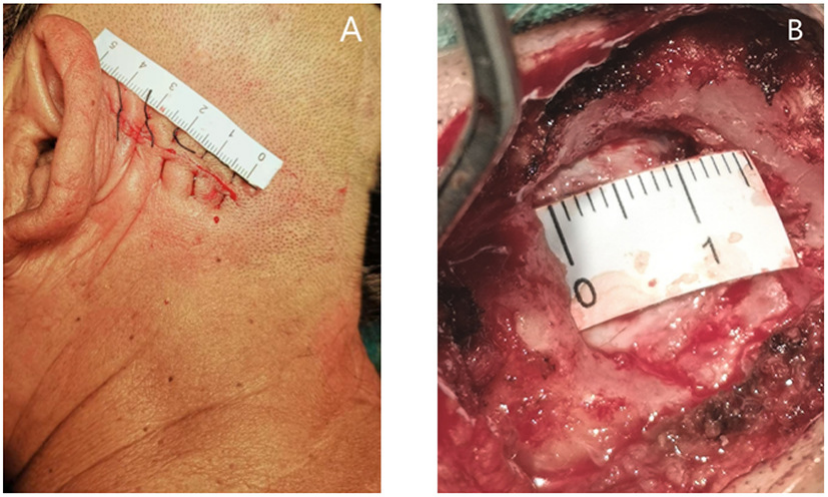
**(A)** Location and length of surgical incision; **(B)** Size of bone window.

For MVD, a healthy lateral position, retrosigmoid approach, 4–5 cm surgical incision behind the ear (Figure 1A), and 1.5–2 cm bone window (Figure 1B) areconsidered. The dura mater is cut to release parts of the cerebrospinal fluid (CSF), and the brain is gently retracted to reduce the field of vision and exposé the surgical area. Attention should be paid to avoiding excessive cerebellar traction which potentially leads to postoperative cerebellar edema and damage to the auditory nerve, resulting in postoperative hearing impairment and other complications (28, 29). The responsible blood vessels are then detected by microscopy, and the blood vessels and nerves are separated using Teflon to achieve decompression. Ideally, the use of Teflon completely opens the blood vessels to achieve sufficient decompression. Finally, the dura mater should be closely sutured during craniectomy, and the open mastoid air chamber should be strictly closed to avoid postoperative CSF leakage (28, 29).

The therapeutic advantages of MVD include higher rates of immediate pain relief and lower rates of complications and recurrence than those of other procedures. The exploration of the mechanism of action and prognostic factors of MVD also provides a theoretical basis for these advantages, and the application of MVD in studies conducted on older populations show that MVD can be used in a wider age range and is safer. Studies comparing MVD with other procedures have demonstrated the advantages of MVD.

## Recurrence and complications

MVD is the only surgical method for managing the etiology of TN, which can mediate remission in 70% of patients with TN experiencing symptoms for ~10 years (30–32). In comparison, MVD does not require nerve damage to achieve a longer duration of remission. The trauma associated with MVD is lower than that associated with other craniotomies, and the hospitalization time is ~1 week. Studies have shown that the satisfaction of patients with early MVD treatment is higher than that of those receiving drug treatment (2, 33–35). Satisfactory pain control rates were 68–88, 65–70, and 44–70% at 1, 5, and 10 years postoperatively, respectively (12, 32, 36, 37). The results showed that patients with MVD had good long-term pain control rates. The recurrence rate was ~14–16% (26, 38, 39). Moreover, the recurrence rate decreased over time (32).

Relevant research has shown that adhesion of Teflon is the most common cause of recurrence after surgery (40, 41). Data on the preferred treatment of patients with recurrent TN are limited. Fossa posterior re-exploration (redo MVD) is an effective treatment measure for recurrent TN after failed MVD (39, 42, 43); however, studies have found that re-MVD is more effective in patients with residual responsible vessels (44, 45). If no vascular compression is found during re-exploration, partial sensory rhizotomy, internal neurolysis, or trigeminal nerve combing can be used (40, 42). MVD combined with internal neurolysis can effectively improve the pain relief rate of patients with recurrent TN. This combination does not increase the incidence of long-term facial numbness or other complications (46). In addition, percutaneous surgery and radiosurgery are good choices for recurrent TN treatment (47). Percutaneous balloon compression (PBC) (48, 49), radiofrequency thermo coagulation (RFT) (50, 51) and gamma knife stereotactic radiosurgery (52, 53) can all achieve ideal treatment effects at the initial stage (Table 2); however, no studies have compared the short- and long-term treatment effects of these three methods.

**Table 2:** Treatment of recurrent TN and pain control rate.

**Operation mode**	**Initial pain relief rate**	**5 years**	**References**
PBC	98.1%	82.7%	Du et al. (48)
RFT	90.2%	51.0%	Lai et al. (50)
GKS	85.0%	56.5%	Kano et al. (52)

Postoperative headache is a recognized complication of the retrosigmoid approach. Its intensity is highest at the initial stage and gradually improves over time (54). Other complications include cerebral infarction or arteriovenous fistula caused by accidental injury of the blood vessels during surgery, hearing loss caused by excessive cerebellar traction and edema, non-rigorous dura mater suture, incomplete mastoid air chamber closure, or CSF caused by poor wound healing (24, 32, 54, 55). Mild complications such as facial numbness, dull sensation, and vertigo, are relatively common. More serious complications, such as facial paralysis, hearing impairment, CSF leakage, anesthesia dolorosa, and infection, are rare, with an incidence of <3%. Mortality is extremely rare, with a reported rate of 0–0.4% in large studies (24, 32, 54–56).

## Principle and prognostic factors

The mechanism of MVD is not completely understood. Some researchers believe that the release of nerve fibers from distorted morphological changes after MVD is the main reason for immediate pain relief (6). The release of fluctuating compression of the responsible vessels reduces spontaneous discharge. Re-perfusion of the previously compressed innervation capillaries and small veins and the innervation edema caused by surgical trauma may further promote the separation of demyelinating fibers in close contact. Remyelination of nerve fibers, improvement of nerve ischemia, and regression of the local inflammatory response may be the reasons for the maintenance of pain relief (6, 57). A small number of patients may have poor surgical results owing to the severe loss of oligodendrocytes, and astrocytes may hinder effective remyelination after decompression (6). Conversely, fibers in close contact after demyelination may have abnormal remyelination before the surgery, which prevents the separation of nerves after decompression (6, 57).

Studies on prognostic factors have shown that severe NVC indicates a good prognosis for MVD (58). Other factors related to a good postoperative prognosis include a shorter course of the disease, type I facial pain, age, and sex (24, 36, 43, 59–61). Moreover, a study showed that purely paroxysmal pain is the only significant predictor of long-term pain relief, which indicates that an early choice of MVD may be more beneficial to patients. Some investigators have reported that MVD, as the first surgical intervention, is preferred over postoperative pain control after MVD treatment through other surgical interventions;(62) therefore, the choice of the first operation also determines the prognosis inpatients to a certain extent.

## MVD in older patients

Whether older patients (>65 years of age) are suitable candidates for MVD remains controversial. Although MVD is a type of craniotomy, it must be performed under general anesthesia, which may not be applicable to some older patients. However, studies have reported no significant difference in the effectiveness and safety of MVD in older patients without surgical contraindication compared to that in young patients (55, 63), and MVD reportedly has a better curative effect in elderly patients. This may be due to the increase in cerebellar atrophy with age, which promotes full exposure of the pontine angle during surgery and improves the decompression success rate (60). Therefore, MVD is a safe procedure for older patients (43).

## Comparison between MVD and other surgical procedures

Surgical treatment of TN includes percutaneous surgery, open surgery, and stereotactic radiosurgery (SRS). Large studies have reported the long-term outcomes of various surgeries. RFT, SRS, and percutaneous balloon compression (PBC) are widely used in addition to MVD.

RFT: RFT has a high immediate pain relief rate; however, the recurrence rate is as high as 46%, and serious complications such as keratitis and corneal hypoesthesia occur. The incidence of other complications such as facial numbness and motor dysfunction is also high (12, 30, 64).

SRS: At present, stereotactic radiotherapy with a gamma knife is the most widely used treatment; however, it takes 6–8 weeks to fully take effect. The initial pain relief rate is lower than that for MVD, and the 5-year recurrence rate is ~25%. The immediate pain relief rate of SRS is equivalent to that of MVD; however, the incidence of long-term recurrence and facial sensation loss is higher (64, 65).

PBC: PBC has become increasingly popular in recent years. Although it is associated with nerve damage, relevant studies have shown that the therapeutic effect of PBC is similar to that of MVD (64, 66). The incidence and types of complications are lower than those of MVD, the operation time is short, the anesthesia risk is small, and the procedure can be repeated (67, 68). However, the incidence of trigeminal motor function damage is high (66%), and repeated surgeries are often required because of the high recurrence rate (64, 69). In addition, older age and postoperative facial numbness are predictive factors for a good prognosis of PBC (70).

## Possible improvement of MVD by endoscopic techniques

**Figure 2:**
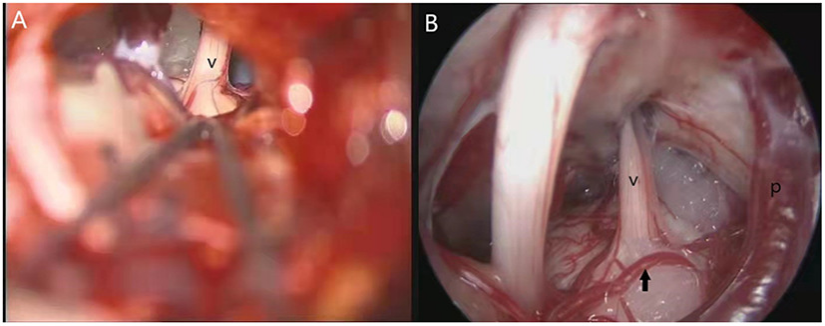
Two pictures from the same operation. **(A)** Visual field of microscope; **(B)** Visual field under endoscope; v, trigeminal nerve; p, petrosal vein; arrow, a responsible artery not found under the microscope.

MVD is the preferred surgical method for CTN; however, being an open surgery, there are also some disadvantages, such as limitations of the microscopic field, surgical wound, occurrence of postoperative CSF leakage, postoperative dizziness, and headache. In recent decades, with the increasing application of endoscopic technology, the above problems have been alleviated to some extent, and related studies have reported that complete endoscopic vascular decompression (EVD) can achieve the same therapeutic efficacy as conventional MVD (38, 54). In addition, because the surgical incision is shorter, the range of muscle peeling and scalp incision is smaller, and the cerebellar retraction is reduced. The incidence of postoperative headache, hearing loss, and other complications is significantly lower than that after conventional MVD (54, 71). Most importantly, in conventional MVD, the field of the microscope is severely limited, and it is difficult to identify the responsible vessels on the dorsal nerve (72). However, endoscopy provides a more optimized visualization effect for MVD; all possible pathogenic vessels can be found (Figure 2) (38, 54, 73), and the detection rate of NVC is more reliable, avoiding insufficient decompression caused by missing the responsible vessels (74, 75). The improvement in the surgical field makes surgeons more confident about the success rate of the operation. Additionally, when the responsible vessel is the vertebrobasilar artery, the operation is more difficult owing to its higher tension. However, the application of endoscopic technology can allow more accurate Teflon placement, achieve more effective decompression, and obtain favorable results in patients with vertebrobasilar TN (76). A recent prospective study reported that EVD has a better long-term treatment effect (77). Their results showed that the increase in fractional anisotropy values and the decrease in apparent diffusion coefficient values in the endoscopic group were greater than those in the microscopic group (77). These two imaging indicators reflecting neuropathy were close to the normal level, suggesting that EVD could improve the microstructure of the trigeminal nerve more effectively. Therefore, the advantages of EVD include shorter operation time, less surgical injury (external or internal), more effective surgical field of vision, and lower recurrence and complication rates (38, 72). However, the premise of these improvements is that operators must be proficient in using endoscopes. Compared with the simplicity of microscopic surgery, endoscopic surgery requires higher proficiency and is more difficult to master. Therefore, efforts are required to promote endoscopic technology in the future (38).

## Discussion

### Before surgery

**Figure 3:**
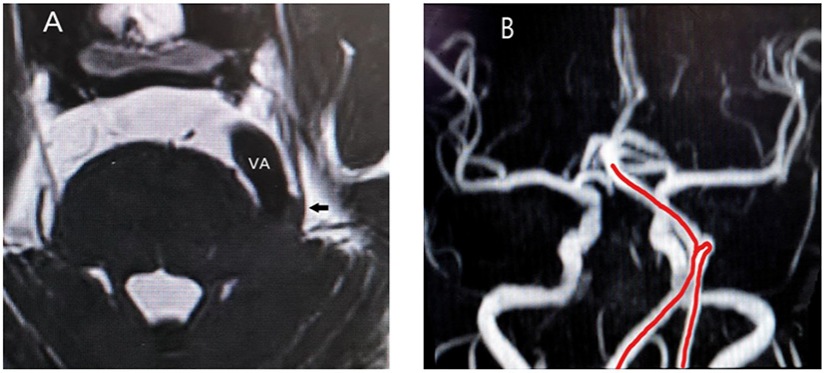
**(A)** Relationship between trigeminal nerve and responsible vessel in a patient on sequences-3D-SPACE; VA, vertebral artery; arrow, trigeminal nerve; **(B)** The red line is the abnormal routing of vertebral artery on magnetic resonance angiography.

The occurrence and development of CTN involve many conditions, and it is a progressive process that has serious negative physiological and psychological impacts on the patients. NVC and the subsequent pathological changes caused by NVC, such as nerve ischemia, demyelination, and compensatory changes of the CNS in response to chronic pain, may be involved in the course of TN. MVD is currently the best surgical treatment, and its choice should rely on preoperative MRI examination, as none of the clinical features can differentiate between the three types of TN. Moreover, since the surgical field of vision is focal, it is impossible to observe the impact of the vascular motion amplitude after effective decompression, while magnetic resonance angiography can fully reveal the overall direction of the responsible vessels before surgery (Figure 3), so that the surgeon can fully prepare.

### During surgery

For the identification of the responsible blood vessels during operation, when the vertebrobasilar artery is the responsible blood vessel, high blood vessel tension is present, with strong pulsation, complex distortion, and difficulty in movement, and the incidence rate is 2–6% in patients with CTN (78). If necessary, Teflon is used for multi-point decompression. The focus is on decompression at the maximum stress point to maximize the recovery of the natural course of the nerves. This is also the key to the success of the MVD. Concurrently, the abductive nerve is protected to avoid postoperative abductive nerve dysfunction. However, the petrosal veins often influence the surgeon's operation. It is advisable to protect the normal blood vessels as much as possible and sacrifice the petrosal veins if necessary (79, 80). The neurological aspects are as follows: studies have shown that the peripheral distribution of pain in patients with TN is related to the location of the NVC, pain in the V1 area is more related to the NVC occurring in the medial and superior position of the trigeminal nerve root, and pain in the V3 area is more related to the NVC occurring in the inferior position of the trigeminal nerve root (81). Therefore, if no obvious responsible blood vessel or branch area of pain is found during the operation, surgeons can sort the corresponding nerve parts according to the peripheral distribution of pain during the operation, which may help achieve certain results.

### After surgery

On the one hand, the existing literature has clarified that severe NVC is a good prognostic factor for MVD. The higher the degree of vascular nerve compression, the more relevant the pathological mechanism of TN is to NVC. Therefore, the effect of MVD on NVC surgery is improved. On the other hand, although procedures such as radiofrequency ablation, gamma knife, and PBC are still widely used, the complications such as facial numbness caused by them are intolerable to patients in severe cases. Although MVD also has its deficits, the addition of endoscopic techniques has improved vascular decompression in many aspects, including a smaller incision and a wider view of the surgical area, reducing pain, and providing more convenience for the surgeon (54, 71).

## Conclusions

The pathophysiology of CTN is not unitary. Over time, vascular compression leads to a series of deeper changes such as local inflammation, nerve ischemia, edema, and CNS alterations, which make drug treatment of CTN difficult. MVD is a surgical treatment for the etiology of CTN. MVD has more prominent short- and long-term treatment effects than other surgical methods, and its selection depends on the precise diagnosis of NVC by preoperative imaging. Moreover, imaging plays an increasingly diverse role in MVD, not only in identifying responsible vessels but also in determining the prognosis and as a tool for scientific research. With the application and maturity of endoscopic technology, vascular decompression may provide patients with TN with beneficial treatment options.

## Author contributions

GY: conceptualization, investigation, methodology, and writing–original draft preparation. JL: conceptualization, writing–review, and editing. YX: writing–reviewing and editing. HX: supervision and reviewing. FM: supervision. All authors contributed to the article and approved the submitted version.

## Conflict of interest

The authors declare that the research was conducted in the absence of any commercial or financial relationships that could be construed as a potential conflict of interest.

## Publisher's note

All claims expressed in this article are solely those of the authors and do not necessarily represent those of their affiliated organizations, or those of the publisher, the editors and the reviewers. Any product that may be evaluated in this article, or claim that may be made by its manufacturer, is not guaranteed or endorsed by the publisher.
